# COVID-19 and older people’s wellbeing in northern KwaZulu-Natal – the importance of relationships

**DOI:** 10.12688/wellcomeopenres.17841.5

**Published:** 2025-05-20

**Authors:** Thabang Manyaapelo, Anita Edwards, Nondumiso Mpanza, Samukelisiwe Nxumalo, Zama Nxumalo, Ntombizonke Gumede, Nothando Ngwenya, Janet Seeley

**Affiliations:** 1 Social Science, Africa Health Research Institute, KwaZulu-Natal, South Africa; 2 Institute for Global Health, University College London Medical School, London, WC1E 6BT, UK; 3 Department of Global Health and Development, London School of Hygiene & Tropical Medicine, London, WC1H 9SH, UK; 4 School of Nursing and Public Health, University of KwaZulu-Natal, Durban, 4013, South Africa

**Keywords:** ageing, infection, non-pharmacological measures, COVID-19, SARS-CoV2, relationships, family, South Africa

## Abstract

**Background:**

The COVID-19 pandemic and the non-pharmacological prevention methods have affected the wellbeing of older people. In this paper we focus on the wellbeing, and vulnerability, of older people in rural northern KwaZulu-Natal, South Africa during the first year of the pandemic.

**Methods:**

We conducted monthly in-depth interviews for up to four months with 26 people aged 57 years and older. A total of 86 interviews were conducted by telephone, because of restrictions on face-to-face contact, and digitally recorded. After transcription and translation, the data were coded thematically, with analysis guided by a wellbeing theoretical framework.

**Results:**

Having access to food, to healthcare and to somewhere they felt safe to stay, was essential for everyone. For many managing expenses became more of a struggle as adult children who had lost their source of employment came home to stay. However, despite the shortages of money, the importance of relationships, whether they are familial or the close community of neighbours, was highlighted in the accounts of many participants. Older people not only got help with day-to-day life from others, but also found solace in the company of others. The sense of community, from family and neighbours, helped to ease some of the stress experienced because of the lockdowns.

**Conclusions:**

The COVID-19 pandemic and the restrictions imposed to limit the spread of the virus impacted the wellbeing of older adults in rural KwaZulu-Natal. Our findings show how the importance of relationships with family and friends contributed to nurturing wellbeing for older people.

## Introduction

Writing in April 2020, Esther Choo (2020) talks of the societal fault lines that COVID-19 has exposed, concluding that once the pandemic has passed, we should remember `how intimately our fates are interconnected, even when we don’t have a virus to bring the point home’ (p. 1333). Our fates are indeed interconnected in a pandemic where an airborne pathogen can be shared from one person to another in a short space of time, if no preventive measures are taken; but those links, our connectedness, have been, and continue to be, felt in many other ways. We begin with an account of the experience of Gogo, aged 88 years, during the first year of the COVID-19 pandemic:

*Gogo lives with her daughter in a homestead in uMkhanyakude District, in northern KwaZulu-Natal Province, South Africa. Her son moved back home after he lost his job when the lockdowns started in March 2020. Gogo has hypertension and diabetes which has made it difficult for her to walk. She relied on her family members to collect her medication from the local clinic. Her grandson helped to collect her monthly old age government pension and he also bought groceries for her. During the lockdown she, her grandson and another of her daughters fell ill with COVID-19 but all recovered. Sadly, another family member, who does not stay with her, who was looking after others who were sick, caught COVID-19 and died. Gogo was sad to not be with the wider family to bury their dead. She missed the visits from the members of her church, which stopped when the pandemic struck. She commented how much people missed being able to go to church, to share and pray together.*

In this paper we explore the impact of the COVID-19 pandemic on the wellbeing of older people, like Gogo, in rural northern KwaZulu-Natal. We focus on the wellbeing, as well as the vulnerability, which come from their connections and relationships.

`Human lives are typically embedded in social relationships with kin and friends across the life span. Social regulation and support occur in part through these relationships […]. The misfortune and the opportunities of adult children, as well as their personal problems, become intergenerational.’ ( Elder, 1994, p. 6)

Our lives are linked to others across our life-course, as Elder reminds us here. Those links move beyond the family and span friends, co-workers, others in our community, our region and country. COVID-19 has lain those links bare both as strengths but also as risks to wellbeing ( Team & Manderson, 2020). We use the word ‘strength’ in this paper to refer to the qualities that promote positive cognition, emotions and behaviour ( Park *et al.*, 2004).

 While the connections between people, for example, of a family staying in cramped conditions together, have been of concern in the biomedical literature because of the mode of spread of the pathogen, there has been a particular focus on the vulnerability of ‘risk groups’ such as older people ( Biswas *et al*., 2021; Brooke & Jackson, 2020; Hassan-Smith *et al*., 2020; Mueller *et al*., 2020). Throughout the pandemic there has been considerable concern about the vulnerability of older people to severe illness and death as a result of SARS-CoV2 infection, with good reason: analysis of excess death data for 2020 for South Africa showed that two thirds of these deaths were in people over the age of 60; with the highest death rate among those aged 60–69 years ( Bradshaw *et al*., 2021). Levasseur *et al*. (2022) refer to ‘situations of vulnerability’ which focuses on ‘a set of circumstances in which one or more individuals experience, at a specific moment in time, one or multiple physiological, psychological, socio-economic or social difficulties’ which increases their risk of being harmed or being unable to cope with the challenges they face (p.275). During the pandemic a number of factors came together to create ‘situations of vulnerability’ for older people.

The risk of severe morbidity and mortality amongst older people was recognised early in the pandemic ( Jordan *et al*., 2020; Wolff *et al*., 2021), but so too were other forms of vulnerability: social, psychological and economic ( Manderson & Levine, 2020). Of particular concern was isolation, and risks to care because of distance from kin and limited access to health services ( Lloyd-Sherlock *et al*., 2020). There also emerged discussion of other risks, other areas in which vulnerabilities were exposed, for different age groups – not only older people -- because of financial, mental as well as physical challenges ( Napier, 2020; The Lancet, 2020). Indeed, some argued that older adults should not be labelled as vulnerable only on the basis of medical criteria, given they have greater psychosocial strength because they have `lived through past challenges in their own lives and having lived through many decades of historical time’ ( Lind *et al*., 2021, p. 47) and, as a result, are less vulnerable to some areas of risk than younger people; Carstensen *et al*. (2020) refer to this as older people’s `emotional experience’.

A focus on specific age groups of people being particularly vulnerable to different threats from the pandemic diverts attention away from the relationships between those people of different ages, people like Gogo described above, and their interdependence and the ways in which situations of vulnerability and strength may be shared. The value of interpersonal relationships – the value placed on recognising `linked-lives’ – is a part of the African philosophical framing of Ubuntuism, found in most of East and southern Africa which provides a set of values and practices to live by ( Ramose, 1999). Ubuntu is the southern African word among people classified as Bantu language speakers often translated into English to mean ‘I am because you are’, stressing the connection of one person to another ( Mbiti, 1969). Ubuntu as a philosophical paradigm as said to include the nature of social reality (ontology), way of knowing (epistemology) and ethics and value systems (axiology) ( Chilisa, 2012). Ubuntuism provides a way in which people see the world and their connections to others and how they interact with their material and metaphysical realities as interdependent beings. Because Ubuntu is seen as rooted in the community and in relationships Chigangaidze *et al*. (2021) call for people to embrace Ubuntu as a guide to the social and psychological response to COVID-19. They note that the emphasis that Ubuntu places on interconnectedness, on relationships, highlights a need to protect each other from infection, but also to respond ‘with generosity, caring and consideration towards others’ (p. 6) when they are in need, and to recognise the ways in which social isolation can harm a person, mentally, physically and socially.

We build from these concerns about social isolation and the threat of disease to suggest that reflecting on the situations of vulnerability and strengths of older people, through the lens of wellbeing, allows us to look at the impact of COVID-19, and the measures put in place to manage the spread, not by viewing an age group of people in isolation, in this case older people in northern KwaZulu-Natal, South Africa, but by recognising an older person’s place in a family and a community. As Hoffmann and Metz (2017, p. 159) describe in their discussion of the lessons an ‘Ubuntu ethic’ holds for development theory, ‘our need to take care of others, as much as our need to be cared for, is central to living well.’

### Ageing and wellbeing

The process of ageing and the pressures caused by life events have multiple interrelated implications for older people’s health and general wellbeing. There is no single definition of wellbeing but there is some consensus that wellbeing includes a general satisfaction with major components in one’s life such as work, having more positive emotions compared to negative feelings or moods and having an overall life satisfaction that encompasses social relationships ( Diener, 2000; Ryff & Keyes, 1995).

Building from Ubuntu, an ethic `which prizes relationships’ ( Ewuoso & Hall, 2019, p. 101), we were guided in our thinking by a wellbeing framework that offers a holistic definition of wellbeing, encompassing three key dimensions: material, relational and the subjective/human components ( White, 2010). Other frameworks focus more on individualism and subjective wellbeing ( Das *et al*., 2020; Diener, 2000; El-Krab *et al*., 2022). The **material** **dimension** comprises assets, welfare, and standards of living. The **relational dimension** is divided into two, social relations and access to public goods; and the **subjective/human dimension** includes capabilities, attitudes to life, and personal relationships. Each of the three dimensions is in turn sub-divided into objective and subjective aspects. The objective dimension examines the tangible objects which make a life better or worse, while the subjective dimension examines how people evaluate the effect of things or actions on their lives. Taking the example of income, the money a person earns can be described as being a part of the objective material dimension of wellbeing, but the satisfaction or dissatisfaction that someone feels about that income is a subjective dimension.

In setting out this framework, White (2010) refers to and emphasizes the role that culture plays in helping to understand how wellbeing is constructed. She notes that wellbeing is grounded in both a particular social and cultural location or context. Culture provides the environment, the context, within which wellbeing develops and shapes not only the way people talk or behave, but also how they think about their needs and wants and assess the material dimensions of wellbeing. This also underlies the values and beliefs they may hold ( Thin, 2018). The cultural context influences how people relate to each other, who lives with whom, how goods are distributed, and needs are met ( White, 2015). To know something about the place where a person lives their life is therefore important in order to understand local beliefs and understandings around wellbeing ( Bond *et al*., 2021).

### The study setting

The social and cultural context of our study, uMkhanyakude District, is in northern KwaZulu-Natal, South Africa. In 2016 the district had a population of 689,090 people, with four percent aged over 65 years old. It is one of the poorest districts in South Africa ( Fransman & Yu, 2019). About 22 percent of the population has access to piped water, and 10 percent of households live within 15 minutes travel time (driving) to a health clinic ( Sharman & Bachmann, 2019). Most people in the area access health services through the public sector District Health System, which includes one referral hospital and 17 primary care clinics. Only five percent of the population holds medical insurance, which results in the majority of people being dependent on the public sector hospital and clinics for healthcare ( McIntosh *et al*., 2021). The level of HIV prevalence in 2018 was 40 percent ( Gareta *et al*., 2021). In addition, there are high rates of non-communicable diseases in adults in the population ( Wong *et al*., 2021). Most households depend on small holder agriculture, state grants and remittances from migrant members of their families. The unemployment rate in the area is high, with 58 percent of adults with no formal employment ( Wong *et al*., 2021). 

Although South Africa is a democratic republic, the district is dominated by traditional structures, which inform and shape the local value systems and norms ( Beall *et al*., 2005). These structures include local councils overseen by AmaKhosi and Induna (traditional leaders who are the AmaZulu King’s representatives in the community) who preside over defined areas and oversee the community.

The study from which the data are drawn for this paper was located within the Population Intervention Programme Demographic Surveillance Area (PIPSA) of the Africa Health Research Institute (AHRI), in uMkhanyakude District. In mid-2018, the population of the PIPSA was estimated to be 140,000 individuals living in approximately 20,000 households ( Gareta *et al*., 2021). The majority of the people living in this area identify as belonging to the AmaZulu ethnic group, sharing customs and cultural practices. These cultural practices include isiZulu traditional rituals for the rite of passage at coming of age (on reaching puberty), for weddings, for funerals and for communicating with ancestors during these events to ask for blessings. These rituals are usually marked by social gatherings, including sharing of food and celebrations to mark the occasion.

An important part of the setting for this paper was the trajectory of the pandemic in South Africa up to the end of our data collection in March 2021. We describe that briefly in the next section.

### COVID-19 in South Africa

In South Africa a ‘national state of disaster’ was declared due to the COVID-19 pandemic with lockdown regulations proposed according to the Disaster Management Act: alert levels one (normal activities) to five (drastic measures taken to contain the virus). From 26 ^th^ March 2020, a 21-day national lockdown at the highest alert level of five was implemented. The lockdown was extended until 30 ^th^ April 2020. The imposed restrictions included closures of businesses classified as non-essential, closures of schools, tertiary institutions and the sale of tobacco and alcohol was suspended during this period. There were also restrictions on large gatherings and the movement of people: people were expected to stay in the location where they lived, with movement for essential work requiring a permit. Non-pharmacological interventions were put in place which included physical distancing, wearing face masks in public places, establishing quarantine sites for people with the infection, promoting regular handwashing and running community education campaigns. The restrictions were eased in stages and this was guided throughout by the infection rates as reported daily by the public health authorities. By the end of August 2020, the country was under ‘level 2’ of the lockdown, with movement allowed but continued physical distancing and mandatory use of face masks in public spaces.

 In December 2020, a second wave of COVID-19 hit the country, and lockdown was again imposed on 29 ^th^ December 2020, which was eased on 1 ^st^ March 2021. The period of our data collection coincided with the opening up of the country after the first wave, and the reimposition of lockdown measures in response to the resurgence of cases. Therefore, the lockdown measures and the waves of COVID-19 infection form the backdrop to the information participants shared as hopes of a swift end to the pandemic receded and learning to manage life with this new infection became accepted as a necessary accommodation everyone needed to make. AHRI staff have been working in the study area for over 20 years, including conducting research with older people. When the restrictions on movement were put in place the Institute staff were able to build on this long standing relationship to investigate the impact of the COVID-19 pandemic on the wellbeing of older people in the area.

## Methods

We conducted a series of up to four monthly in-depth interviews with 26 people aged 57 years and older. The aim was to investigate how the COVID-19 pandemic affected the wellbeing of these older people during the period between October 2020 and February 2021. The interviews, which were conducted by telephone because of restrictions on face-to-face contact, took the form of an oral diary, with the participant sharing with the interviewer what had been happening in their life over the previous week – a recall period that we had found to be appropriate in other studies using this method with older people ( Wright *et al*., 2012). During the first interview, the participant was invited to share their experience of the first six months of the pandemic (from March 2020) and the associated periods of lockdown. The inclusion criteria for our participants included the following: 1) over the age of 55 years, 2) had access to a mobile phone, 3) had adequate hearing levels to be able to hear the interviewer and follow the conversation, 4) willingness to participate in at least four interviews over a six-month period. These criteria were developed to consider the constraints imposed on data collection during the pandemic.

A randomly selected list of eligible participants with contact details in the database was extracted by a PIPSA data manager for research assistants to recruit into the study. An effort was also made to select people from across the PIPSA area to ensure a spread of people from more and less remote locations. After each of the calls made to the participants, airtime (time paid for a set amount of mobile phone use) would be loaded onto each of the participants mobile phones as a token of appreciation for their time.

### Ethical approval

Ethical approval for this project was obtained from the University of KwaZulu-Natal Biomedical Research Committee in South Africa (BREC Ref No: 00001642/2020) and the London School of Hygiene & Tropical Medicine Ethics Committee (Ref. 22666). All methods, including oral informed consent, were performed in accordance with the relevant guidelines and regulations. All participants gave verbal consent to participate in the study. Each participant was asked to confirm their name, surname and identification number or their date of birth to the interviewer and asked to repeat the phrase, ‘Yes, I consent to participate’ or ‘No, I do not consent to participate’. The ethics approval made provision for verbal consent especially since this was during the imposed lockdown and physical contact was discouraged. All names used in this paper are pseudonyms.

### Data collection

All participants had experience of the regular demographic and health surveillance conducted by staff from AHRI, and some had taken part in a study with older people in 2018, so they were familiar with the research organisation. For the recruitment into this study, two experienced social science research assistants, fluent in isiZulu the local language, rang prospective participants and explained the purpose of the study, and invited people to take part. If the person was interested in participating, they then sought their consent to participate in the study. During this initial call, an appointment was made for the actual interview, and for some people who were eager to move forward that appointment followed immediately. The same two social science research assistants conducted all the interviews in isiZulu over the telephone.

The interviews lasted between thirty minutes and one hour. A topic guide ( Manyaapelo *et al.*, 2022b) was used as an aid to guide the discussion over the time period covered in the interview to reflect on any physical changes and social changes in the participant’s life over recent times, and also any emotional high points (something that may have made them happy) or low points (when they were sad).

All the interviews were conducted from the AHRI ‘call centre’ established during the pandemic to support remote data collection from a secure and private location. Interviews were conducted using a Mitel IP phone system using a handsfree Single Ear Noise Cancelling headset that automatically recorded the conversation. Participants were encouraged to find a quiet and private location from which to take the call. 

### Data analysis

This study used the framework approach for data analysis ( Gale *et al.*, 2013). This is a systematic, flexible, iterative and interactive method to analyze qualitative data where the whole study data collection team can participate. Data analysis was a 5-step process, 1) transcription and translation, 2) coding and coding framework, 3) analytical memos, 4) thematic matrix, 5) wellbeing framework orientation. All interviews were transcribed and then translated into English. The same people who conducted the interviews were responsible for the transcriptions and translations. Following translations, a senior research assistant conducted quality checks of all the transcripts to ensure that the meaning conveyed in the isiZulu version was retained in the English version. The transcripts were given a unique identity number as a label; names of participants and people they mentioned during the interview, were not included. The label assigned was also used to denote which interview was which in the series, for example *TIDI01* was for the first round of interviews while *TIDI04* was from the fourth round. The coding of the transcripts was managed by a team of two social scientists using NVivo 12 (Open-source alternative software is Taguette 1.3.0). A coding framework was drafted after the first two interviews, as themes emerging from the data were identified and added to those which were drawn from the topic guide (drawn both from the topic guide used and themes emerging from the data). The coding framework was agreed amongst the team, made up of the authors of this paper, before being used to code the data, with the team practicing ‘constant comparison’ to cross check that the coding conducted by different people maintained the same approach to how the codes were interpreted. Analytical memos, by theme, were developed and used as an aid to discussion during debriefing meetings with the principal investigator and wider team. For each of the main themes a thematic matrix was produced using Excel to bring the data on a particular theme together for sharing within the team. This framework approach to analysis allowed us to work together to gain an overview of the information on each theme and to discuss the differences between participants in the information pertaining to particular themes ( Gale *et al*., 2013). Using this approach we could compare and contrast the information emerging from our analysis as well as look for relationships between themes. This is how we began to see `wellbeing’ as an important factor in people’s lives.

The analysis process resulted in seven themes being highlighted that related to the concept of wellbeing. These included: livelihood activities during lockdown, household composition, family traumas, medical needs, methods of (COVID-19) prophylaxis, sources of income and sources of happiness. These themes were then further grouped under the three dimensions of wellbeing: material, relational and subjective. Livelihood activities during lockdown (material), household composition (relational), family traumas (relational/subjective), medical needs (relational), methods of (COVID-19) prophylaxis (subjective), sources of income (material) and sources of happiness (subjective). We present our findings under the three wellbeing dimension headings (material, relational and subjective). In order to improve transparency of this research study, the Standards for Reporting Qualitative Research checklist was completed ( Manyaapelo *et al*., 2022c).

## Results

Twelve men and fourteen women between the ages of 57 and 88 years participated in a total of 86 interviews over a period of four months (see Table 1). It was not possible for all the participants to manage a call every month for four months (which accounts for the shortfall of 18 interviews), but for all those who took part we were able to trace with them their sense of wellbeing across the study months. An anonymized summary of the interviews is available ( Manyaapelo *et al*., 2022a).

**Table 1.** Demographic profile of participants and interviews conducted.

	Sex	Age	Interview 1	Interview 2	Interview 3	Interview 4
1	Male	67	Yes	Yes	Yes	Yes
2	Female	62	Yes	Yes	Yes	Yes
3	Male	66	Yes	Yes	Yes	Yes
4	female	65	Yes	Yes	Yes	Yes
5	Male	69	Yes	Yes	Yes	Yes
6	Male	61	Yes	Yes	Yes	Yes
7	Male	68	Yes	Yes	Yes	Yes
8	Female	74	Yes	Yes	Yes	Yes
9	Female	76	Yes	Yes	Yes	Yes
10	Male	80	Yes	Yes	Yes	Yes
11	Male	75	Yes	Yes	Yes	Yes
12	Male	61	Yes	Yes	Yes	Yes
13	Male	75	Yes	Yes	Yes	Yes
14	Male	75	Yes	Yes	Yes	Yes
15	Female	63	Yes	Yes	Yes	No
16	Female	62	Yes	Yes	Yes	No
17	Male	66	Yes	No	No	Yes
18	Female	74	No	Yes	Yes	Yes
19	Female	63	Yes	Yes	Yes	No
20	Female	57	Yes	No	Yes	No
21	Female	88	Yes	Yes	Yes	No
22	Female	81	No	Yes	Yes	Yes
23	Female	61	No	No	Yes	Yes
24	Female	67	No	Yes	Yes	No
25	Female	71	No	Yes	Yes	Yes
26	Male	68	No	Yes	No	No

**Material dimension** (livelihood activities, sources of income)

Having access to food, to healthcare and to somewhere they felt safe to stay, was essential for everyone. However, some people saw their source of income fall away as the first lockdown was imposed. For example, a man in his 60s had been earning some money taking children to school in his private vehicle, as soon as the schools closed this source of income stopped. His adult children stepped in to support him, and he also got some financial support from his nephews and nieces. The role of an uncle is an important one in local culture, and he was grateful that his nieces and nephews recognized their responsibility by supporting him. He did his best to keep the car in working order during lockdown in case of need by the family, even though he could not get help with repairs at that time. He felt this service was something he could offer the family when there was a need.

For most people in this study, the old age pension received from the government was their primary source of income which was then supplemented by a remittance from their adult children or small retail activities: “ *Yes…yes…we receive the pension grant then we get money from the sugarcane. I also get some cents after I make sales from the mats”* (man, 62 years).

During the period of lockdown, the government had also pledged to give qualifying people the Special COVID-19 Social Relief of Distress amount of R350 ($20) a month. Some of the participants spoke about how they had received this grant while some had spoken about how they were still waiting and there was no indication if they would receive it or not. They mentioned how this money would be used to buy food. 

Additional income was also received in the form of child support grants for those who qualified. Keeping livestock and maintaining vegetable gardens for homestead consumption was a very common thing among most of the people interviewed in this study. For some, the standard of living could be seen as very low. A 67-year-old man described his happiness as being brought about by never running out of food.

“ *What I can say made me happy, you see as from the last time I talked with you, this month we never run out of food in this home, we are eating with the children and we get full”.*

This statement suggests that he was used to not having enough food. The month before we spoke to him, with his family around who could provide food for themselves and for him, they had had enough to eat.

**Relational dimension** (household composition, medical needs, family traumas)

People respond and deal with hardships in different ways. Some people found the strength to cope despite extended periods of isolation from loved ones. A 75-year-old gentleman, Baba, in the initial conversations spoke about how he was living by himself since his wife had been away to look after a sick child. *“Yes, my wife would collect treatment for me at S_ (name of the clinic). When she has given me the treatment she goes back home because she left the child alone, she stays with the child it is just the two of them”* In the later interviews he started to open up about his life circumstances and confided to the interviewer that his wife was in the process of divorcing/leaving him. He showed a strong sense of resilience considering the recent separation from his spouse, which meant he now needed to cook, clean, and buy groceries for himself. *“I clean the house myself; I do the washing myself, I mow the yard, you can see how I planted, you will say no, there are many people in this home, but I am alone”* His adult children had not visited him throughout the lockdown, which left him vulnerable and lonely in his isolation. In the final interview with us, when he had grown to know and trust the interviewer, he expressed his need for company and friendship.

The importance of relationships, whether they are familial, or the close community of neighbours was also highlighted by the 78-year-old woman, Thembi, who was taking care of her sick husband. This caring role restricted her movement and she could not leave her house very much. Her husband subsequently succumbed to his illness and passed away. Some of her adult children who worked as casual labourers came home after their employment ended to stay with their mother. Their mother, Thembi, received an old age pension, which was enhanced for a period of time during the pandemic, which helped the family to manage. The grandchildren who were at home assisted with the household chores and errands. Thembi was on hypertension medication and the neighbour’s son had been helping to refill her prescriptions since he worked at the local clinic. *“I am on BP treatment, my neighbour’s child helped me to refill on my behalf because he works at the clinic, even now he helps me with refills. He started to refill on my behalf because I could not leave my husband alone”* The sense of community, from family and neighbours, helped to ease some of the stress experienced as result of the lockdowns.

A 63-year-old man, Thabo, was approached by their neighbour to ask if they could use his car to drive their adult child, who was mentally unwell, to the hospital because the child was in need of treatment. This happened at night. His car had problems with the lights, so they went together to a second neighbour who had a car but no petrol. They decided to siphon petrol from the car with no lights to the second neighbour’s car so they could use that car. The process took some time, sadly, by the time the car was ready it was too late, the person they were trying to help had committed suicide.

This sense of community and neighbourliness can also be seen in how some small groups of church members would visit some of their members for prayer and communion when the restrictions eased to allow some movement in the open air in the neighbourhood (even though large gatherings in church were still banned). These visitations also served to deliver food parcels to the congregants who missed the opportunity to attend church because of the restrictions.

**Subjective/human dimension** (moral support needs, family traumas, sources of happiness)

A 60-year-old man, Sphe, had initially been sceptical about the pandemic in the first few weeks after the news broke and had not considered that the pandemic would affect him. However, as time went on things changed and he said: *“I started believing it when I started hearing from the radios that people are dying and even now my fear is escalating, I am still very scared”*. He had reason to be scared; at the time he told us this South Africa was experiencing the second wave of infections and people were once again dying from COVID-19 in the community. Other tragedies affected him too: during the pandemic one of his neighbour’s daughters committed suicide and he also had a death in the family (not connected to COVID-19). He was also deeply saddened that he was unable attend funerals, *“So, it is painful, even animals gather if there is death”*. The fact of not being allowed to view the body of the deceased, because he could not attend the funeral, left a deep impression on Sphe. However, for Sphe even during this difficult time, he was still very grateful for those around him and was able to find joy from the support he got from his children and extended family. The importance of supporting relatives and honouring the dead by being present at funerals was often mentioned by the older people. There was a deep sense of unease at missing these important rites of passage. One man told us that he was very distraught when he was unable to attend a funeral of a relative and, as the eldest in the family, he had important cultural duties to perform.

The fundamental need to relate is at the core and driver of human interactions and of particular significance is the personal relationships. These personal relationships help to understand psychological wellbeing. Similar to the situation of Baba (described above), a 66-year-old man, Dumisani, was separated from his spouse. He had been working far away from home and only noticed when it was too late that things were not going well at home. The problems at home arose partly from the loss of his daughter, who was shot and died. All this happened before COVID-19. During the pandemic he stayed with a new partner who would often leave him by himself for extended periods when she went to visit her own family. His partner subsequently contracted COVID-19 and was initially very sick, but later recovered. He spoke about the difficult relationship with his ex-wife, which he said had also strained relations with his children who he rarely saw as result. Dumisani complained of being lonely, especially when his current partner went away. 

Most of the people in this study found things that made them joyous despite the hardship they were living through. The phone call with the study interviewer was one such event, in addition the airtime received from the study as a token of appreciation, was welcomed with much gratitude.

The support received from the adult children in the form of groceries or in some cases money which they sent, was very much appreciated. It was clear that most older people did not have any expectation that their adult children should support them, so when an unexpected gift arrived they were filled with happiness.

Social connectedness can also be cultivated in communion. A celebration of life events usually makes it possible for family and friends to meet and share life experiences. A 63-year-old woman, Thulile, was excited because her daughter had become engaged to be married. These events are marked by slaughtering of animals and the presence of family and friends from far. Thulile observed that:

*“There is nothing that worried me because my daughter’s fiancé came home and asked for a umkhehlo [engagement party] so my daughter will have umkhehlo and memulo [rite of passage celebration].*

In the midst of all the worries over COVID-19, such celebrations lifted people’s spirits.

## Discussion and conclusions

We found that the wellbeing of older people in northern KwaZulu Natal was affected by COVID-19 in a variety of ways, which highlighted the importance of relationships. These relationships are those between older people and their children, their grandchildren, their spouses/partners, their peers, and their community at large. While COVID-19 had impacted on the physical health, income, interaction with family and friends, physical activity and psychological health of older people, some of these effects were alleviated or exacerbated by the relationships the older people had with other people in their lives.

Social relationships have been found to have a significant impact on health and the manner through which this happens can be behavioural, psychosocial and physiological ( Umberson & Karas Montez, 2010) as situations of vulnerability shift. During the lockdown the inability to travel freely affected older people’s wellbeing as they experienced social isolation when they were cut off from friends and the opportunity to attend social gatherings.

Most of the participants in this study did not live with their adult children. The restricted mobility imposed by the lockdowns prevented some of the adult children from going home to the rural area to visit or to be with their parents for extended durations. The support that could have resulted from these visits was removed leaving the older people vulnerable to a range of challenges which affected their wellbeing. Telephone calls may have helped to alleviate some of this stress. Research in the United States has shown that older people who had phone calls with family or friends at least twice a week showed lower odds of a mild cognitive impairment (MCI) or dementia ( Gardener *et al*., 2021). The authors also found that those who were socially isolated and lonely showed increased odds of MCI or dementia compared to those who were socially isolated but not lonely, as well as those who were lonely but not isolated. Social relationships foster social connectedness, which is critical for good mental health and wellbeing in South Africa and elsewhere ( Geffen *et al*., 2019; Luo *et al*., 2020).

Cultural practices and traditions help to orientate and locate social relationships. This is particularly important in a setting such as northern KwaZulu-Natal where the self is understood to be interdependent/collectivist, where individuals are seen as interconnected with others, both the living and the ancestors. Ubuntu becomes central to how relationships are formed and maintained meaning that the life experiences of individuals are told through their relationships with others and their place within their communities ( Okoro, 2015). One of the most important customs that displays this interconnectedness is in death and the days of mourning observed, which culminate with a burial where the family, friends and community congregate. The days leading up to the funeral where the extended family is often present from afar makes it possible to disperse some of the grief and offer comfort. These days of mourning are characterized by communal tasks of food preparation and general preparation of the household to welcome those arriving to pay their respects. The lockdown restrictions prevented people from burying their dead in the customary manner. A sudden death because of COVID-19 was compounded by the inability to perform funerary rights, in South Africa and elsewhere ( Cardoso *et al*., 2020; Simpson *et al*., 2021).

Cultural practice also speaks to the social structure and positioning of the individual by age, gender and social designation (younger father or younger mother etc.). In isiZulu custom, as among other ethnic groups in South Africa, there are roles and responsibilities assigned to these positions ( Sooryamoorthy & Makhoba, 2016). For example, *rangwane* (father’s younger brother), *mangwane* (mother’s younger sister), *rakgadi* (father’s sister) and *malome* (mother’s brother) are automatically assigned parental roles to the children born by their siblings and are expected to fulfill these roles to co-parent the children ( Mokuoane, 2018). This ensures that there is never a parental void in the event a parent, for any number of reasons (away for work, death), is absent. The socialization of children among AmaZulu and other ethnic groups in South Africa, ensures that they are brought up with an appreciation for the role in their parenting of others kinship group their parents. Thus, it was not surprising to hear of the pride and joy an uncle felt in being appreciated by the nephews and nieces, as noted above in our findings, who he would have considered his children. Another man was distraught at not being able to perform his customary role at a relative’s funeral, which highlights the importance of not only caring for the living but also honouring the dead who, as ancestors, remain a part of the family. These examples of linked lives provide examples of the social relationships, which older people, cherish and illustrate a sense of duty experienced in these relationships, either in the family or community, which are very important to wellbeing ( Schatz & Gilbert, 2012).

Previous studies with older people in South Africa emphasized the positive impact of structural factors such as the pension grants on quality of life ( Ralston *et al.*, 2019). However, the growing population of older people is predicted to negatively impact the health budget due to an increased burden of chronic conditions ( Solanki *et al.*, 2019). While structural factors are important, more research is needed to understand the psychosocial factors which affect older people in daily life in South Africa. The findings of this study will assist future research to investigate policies that are more holistic and community centred.

This study has limitations. Due to the lockdown restrictions and to also limit exposing this vulnerable group to infections, all the interviews were conducted by telephone. It was difficult to cultivate a strong rapport over the phone and it is not possible to observe non-verbal cues which can sometimes assist in an interview. There were also instances when the poor mobile phone network connection interrupted some of the interviews thereby making some interviews unnecessarily longer.

The COVID-19 pandemic and the restrictions imposed to limit the spread of the virus and to ultimately reduce morbidity and mortality have impacted the wellbeing of older adults in rural KwaZulu-Natal. We have illustrated the importance of relationships and while some relationships had in some instances caused distress, more often these relationships had been a source of strength to help older people withstand the hardships during the pandemic.

## Consent

Oral informed consent for publication of the participants’ details was obtained from the participants. Oral informed consent was obtained because the study was conducted during the COVID-19 lockdown and the University of KwaZulu-Natal Biomedical Research Committee made provision for verbal consent given the strict lockdown regulations at the time of the data collection. Oral consent was documented by using a Mitel IP phone system using a handsfree Single Ear Noise Cancelling headset that automatically recorded the conversation. 

## Data Availability

Figshare: Underlying data for ‘COVID-19 and older people’s wellbeing in northern KwaZulu-Natal – the importance of relationships’. https://doi.org/10.6084/m9.figshare.19738477 ( Manyaapelo *et al*., 2022a) Figshare: Extended data for ‘COVID-19 and older people’s wellbeing in northern KwaZulu-Natal – the importance of relationships’. https://doi.org/10.6084/m9.figshare.19738507 ( Manyaapelo *et al*., 2022b) Figshare: SRQR checklist for COVID-19 and older people’s wellbeing in northern KwaZulu-Natal – the importance of relationships’. https://doi.org/10.6084/m9.figshare.19738438 ( Manyaapelo *et al*., 2022c) Data are available under the terms of the Creative Commons Attribution 4.0 International license (CC-BY 4.0)
