# Supplementary material for: COVID-19 and older people’s wellbeing in northern KwaZulu-Natal – the importance of relationships
Source: Wellcome Open Res. 2025 May 20;7:168. Originally published 2022 Jun 9. [Version 5] doi: 10.12688/wellcomeopenres.17841.5 (PMC12198728; doi:10.12688/wellcomeopenres.17841.5)
Supplement: Peer review history

# Reviewer report: Reviewer response for version 5

**DOI:** 10.21956/wellcomeopenres.26677.r123862

**Published:** 2025-06-25

**Authors:** Esther Williamson

**Affiliations:** 1 University of Oxford, Oxford, England, UK

I am happy with responses to my previous review and it is suitable for indexing.

Is the work clearly and accurately presented and does it cite the current literature?

Yes

If applicable, is the statistical analysis and its interpretation appropriate?

Not applicable

Are all the source data underlying the results available to ensure full reproducibility?

Partly

Is the study design appropriate and is the work technically sound?

Yes

Are the conclusions drawn adequately supported by the results?

Yes

Are sufficient details of methods and analysis provided to allow replication by others?

Partly

Reviewer Expertise:

Physiotherapy, qualitative research, randomised controlled trials, older people

I confirm that I have read this submission and believe that I have an appropriate level of expertise to confirm that it is of an acceptable scientific standard.

# Reviewer report: Reviewer response for version 4

**DOI:** 10.21956/wellcomeopenres.24776.r107920

**Published:** 2024-11-20

**Authors:** Esther Williamson

**Affiliations:** 1 University of Oxford, Oxford, England, UK

A very interesting study exploring the experiences of older people during the COVID-19 pandemic who are living in a rural area of South Africa. The authors provide a comprehensive description of the area to give context to the study.

 Page 6 – “From 26th March 2020, a 21-day national lockdown at the highest alert level of five was implemented. The lockdown was extended until 30th April 2022” Is April 2022 correct?

 Study inclusion/exclusion criteria – please state clearly the inclusion and exclusion criteria for participants in your study. This is not clear. For example, you mention an age criteria but not what the criteria was. Page 6. It looks like additional participants were recruited after the first interviews were done. Why was this?

 You say participants were randomly selected by a PIPSA data manager. Did you have a database of people living in the area? Please explain how this was done. From how many people was your sample selected?

 Data analyses – what method of data analyses was used? This should be stated at the start of the analyses section. Was it a framework analyses? And should be referenced – I see this is done later on in the paragraph.

 What processes were put in place to check the trustworthiness of the analyses?

 The findings are summarized clearly, but one section (relational dimension) has no supporting quotes and I notice previous reviewers have asked that these be included. This would improve the quality of the paper.

 Discussion – what are the implications of the findings of this study? For example is there a potential impact on public policy or areas of further research? The authors should include this in the discussion.

Is the work clearly and accurately presented and does it cite the current literature?

Yes

If applicable, is the statistical analysis and its interpretation appropriate?

Not applicable

Are all the source data underlying the results available to ensure full reproducibility?

Partly

Is the study design appropriate and is the work technically sound?

Yes

Are the conclusions drawn adequately supported by the results?

Yes

Are sufficient details of methods and analysis provided to allow replication by others?

Partly

Reviewer Expertise:

Physiotherapy, qualitative research, randomised controlled trials, older people

I confirm that I have read this submission and believe that I have an appropriate level of expertise to confirm that it is of an acceptable scientific standard, however I have significant reservations, as outlined above.

# Response

**Published:** 2025-04-26

**Authors:** Thabang Manyaapelo

**Affiliations:** Africa Health Research Institute, South Africa

REVISED - COVID-19 and older people’s wellbeing in northern KwaZulu-Natal – the importance of relationships *Thank you for the insightful and constructive comments.* **Reviewer 3** **Comment 1:** A very interesting study exploring the experiences of older people during the COVID-19 pandemic who are living in a rural area of South Africa. The authors provide a comprehensive description of the area to give context to the study.

 Page 6 – “From 26th March 2020, a 21-day national lockdown at the highest alert level of five was implemented. The lockdown was extended until 30th April 2022” Is April 2022 correct?

**Response: Thank you. This date has been corrected to April 2020** **Comment 2:** Study inclusion/exclusion criteria – please state clearly the inclusion and exclusion criteria for participants in your study. This is not clear. For example, you mention an age criteria but not what the criteria was. Page 6. It looks like additional participants were recruited after the first interviews were done. Why was this? **Response:** **Thank you. The inclusion criteria were included in the Methodology section and read as follows:** **The inclusion criteria included the following: 1) over the age of 55 years, 2) had access to a mobile phone, 3) had adequate hearing levels to be able to hear the interviewer and follow the conversation, 4) willingness to participate in at least four interviews over a six-month period.** **No, we did not recruit additional participants after the first interviews. Not all the participants who were recruited for the first interview were able to participate in the interview at that time. The same participants were called again for the subsequent interviews. This means that for all four interviews scheduled for each participant, the majority were available for all four.**

**Comment 3:** You say participants were randomly selected by a PIPSA data manager. Did you have a database of people living in the area? Please explain how this was done. From how many people was your sample selected? **Response:** **Under the heading “Study setting” in paragraph 3 we describe the Population Intervention Programme Demographic Surveillance Area (PIPSA) database of the Africa Health Research Institute (AHRI), in uMkhanyakude District. In mid-2018, the population of the PIPSA was estimated to be 140,000 individuals living in approximately 20,000 households.** **The following sentence has been edited for clarity:** ***A randomly selected list of eligible participants with contact details in the database was extracted by a PIPSA data manager for research assistants to recruit into the study.***

**Comment 4:**

 Data analyses – what method of data analyses was used? This should be stated at the start of the analyses section. Was it a framework analyses? And should be referenced – I see this is done later on in the paragraph.

**Response:** **The following sentence was added at the start of the paragraph to state the method of analysis.** ***This study used the framework approach for data analysis (Gale, et al, 2013).  This is a systematic, flexible, iterative and interactive method to analyze qualitative data where the whole study data collection team can participate.*** **Comment 5:**

 What processes were put in place to check the trustworthiness of the analyses? **Response:** **Thank you. The framework approach we used in this study is designed to include credibility and confirmability, which are some of the key pillars of trustworthiness. We had regular peer debriefs and reflexive journaling which aided in our analysis process.**

**Comment 6:**

 The findings are summarized clearly, but one section (relational dimension) has no supporting quotes and I notice previous reviewers have asked that these be included. This would improve the quality of the paper. **Response:** **Thank you. Quotations which relate to the relational dimension of the analysis were added to the text as suggested.**

**Comment 7:** Discussion – what are the implications of the findings of this study? For example is there a potential impact on public policy or areas of further research? The authors should include this in the discussion. **Response:** **We have revised our discussion to include a paragraph on the potential impact on public policy and future research and included the references in the Bibliography.**

# Reviewer report: Reviewer response for version 4

**DOI:** 10.21956/wellcomeopenres.24776.r87197

**Published:** 2024-07-15

**Authors:** Elfriede Derrer-Merk

**Affiliations:** 1 University of Liverpool, Liverpool, UK

**COVID-19 and older people’s wellbeing in northern KwaZulu-Natal – the importance of relationships**

  4 ^th^ feedback

 Dear authors,

 this is the fourth feedback on this manuscript, and I would suggest reading and addressing the previous versions of my recommendations. As far as I could see in the new version, the authors did not address the other aspects repeatedly demanded, except for providing a demographic table and recalculating the number of participants.

 As the authors refuse to address the trustworthiness of the data management (e.g., inconsistent labelling, the naming of participants, and numbering in the demographic table and the table provided as Excel files do not match), explain why they recruited more participants in the second round of interviews, add more evidence (quotes) to the findings (requested also from reviewer 2), and explain the purpose of the longitudinal approach and what the findings were, as suggested in review 3, I decided to reject the paper.

 Furthermore, I would suggest not using the classification of resilience if you don’t provide the definition, you are drawing on. This concept is contested and needs further thoughtful consideration if used.

 Best wishes Elfriede Derrer-Merk

Is the work clearly and accurately presented and does it cite the current literature?

No

If applicable, is the statistical analysis and its interpretation appropriate?

Not applicable

Are all the source data underlying the results available to ensure full reproducibility?

No

Is the study design appropriate and is the work technically sound?

Partly

Are the conclusions drawn adequately supported by the results?

Partly

Are sufficient details of methods and analysis provided to allow replication by others?

No

Reviewer Expertise:

Gerontology, COVID-19 pandemic research, qualitative methodology, risk communication, cross-cultural studies

I confirm that I have read this submission and believe that I have an appropriate level of expertise to state that I do not consider it to be of an acceptable scientific standard, for reasons outlined above.

# Reviewer report: Reviewer response for version 3

**DOI:** 10.21956/wellcomeopenres.23492.r79127

**Published:** 2024-05-03

**Authors:** Gabriella Meltzer

**Affiliations:** 1 Columbia University Mailman School of Public Health, New York, USA

Thank you for the opportunity to review the study, "COVID-19 and older people's well-being in northern KwaZulu-Natal - the importance of relationships." I appreciate that the authors contributed to the qualitative literature on the non-biomedical vulnerability of older adults during COVID-19, particularly in a LMIC and rural setting when most studies have taken place in higher income settings. The authors argue, and I agree, that it is important to consider older adults’ wellbeing holistically, beyond simply prophylactic measures to protect them from severe morbidity and mortality from the COVID-19 virus itself. The authors do a nice job of grounding the paper in extant literature and relevant, culturally appropriate theory. They framed their paper and organized their results around the southern African philosophy of Ubuntuism, or “linked lives,” which is comprised of the material dimension, relational dimension, and subjective/human dimension, in order to describe older adults’ wellbeing during periods of lockdown in rural South Africa. I feel that the authors gave the reader of a good sense of the study setting, did a good job describing their methodology, and the results were organized in a nuanced way. 

 I have no major comments for improvement at this time. The authors did a nice job of incorporating previous reviewers’ comments.

 Minor comments: 

 -I would recommend that the authors include a table of the sociodemographics of the study participants

 -At the end of the Introduction, the authors should say 29th December 2020 rather than 2021

 -I feel that the results section would be strengthened with more direct quotes from participants

Is the work clearly and accurately presented and does it cite the current literature?

Yes

If applicable, is the statistical analysis and its interpretation appropriate?

Not applicable

Are all the source data underlying the results available to ensure full reproducibility?

Yes

Is the study design appropriate and is the work technically sound?

Yes

Are the conclusions drawn adequately supported by the results?

Yes

Are sufficient details of methods and analysis provided to allow replication by others?

Yes

Reviewer Expertise:

Qualitative research; disaster studies; life course

I confirm that I have read this submission and believe that I have an appropriate level of expertise to confirm that it is of an acceptable scientific standard.

# Reviewer report: Reviewer response for version 3

**DOI:** 10.21956/wellcomeopenres.23492.r76533

**Published:** 2024-04-13

**Authors:** Elfriede Derrer-Merk

**Affiliations:** 1 University of Liverpool, Liverpool, UK

**COVID-19 and older people’s wellbeing in northern KwaZulu-Natal – the importance of relationships**

 Dear authors, thanks for clarifying further some aspects of your study.

 However, some questions are not answered yet, please see below.

 I asked to clarify the number of participants who took part in each interview over four months. You added some information but it is still unclear. Please see the attached information in the files section below.

 I made the effort to create a table myself to see who was interviewed and at which point, where the 17 interviews could not be conducted. Having a total of 87 interviews 14 participants took part in all four interviews, one participant only once. This information is experienced as confusing and messy. Despite asking for clarity your representation does not give a sufficient overview.

 I coloured the participants labelling in the four interviews who did not take part in all interviews to see where the shortfall is coming from. Unfortunately, this was difficult. I would recommend the authors make it clearer. Additionally, the transparency of the sample and design of the study (longitudinal?) aims to provide trustworthiness of the analysis as an important quality criterion for qualitative research.

 Unfortunately, the given page numbers in the revised version do not match when the document is accessed via PDF.

 I understand why the longitudinal approach is important as this design looks at changes over time. But this is not reflected in the feedback nor the latest version. Given that 14 participants took part in all 4 interviews, 1 only once, according to the provided tables, some participants did not join the first or the last interview see my table above. Did the authors find any changes over time how could this be related to lockdown or easing the lockdown?

 This data collection raises the question of why the authors added more participants to their sample after the first round of interviews. When was theme saturation reached? The question of trustworthiness and how the data was handled arose.

 Additionally, the labelling does not provide transparency: the participants' names are pseudonyms in the manuscript, that’s fine; then the labelling has been described including the numbering of the interview this would be fine as well but the output matrix shows some different labelling. If the authors have concerns about anonymity this can be assured with various methods, in this manuscript it is not clear how they are connected with the provided output matrix.

 Please see my table below [in files] and the description in the manuscript:

 Response: The label assigned was also used to denote which interview was which in the series, for example TIDI01 was for the first round of interviews while TIDI04 was from the fourth round.

 The authors added information about the disaster measures and broad information on how the interviews were related to the lockdown, but a timeline Figure would have helped to understand the circumstances much better.

 Page 10 are references so I can’t really see the changes.

 I asked for more evidence based on the data instead of interpretation to enhance the trustworthiness of the analysis. The history that qualitative research was long seen as not scientific with low scientific quality, should be pushed back by having criteria for quality and trustworthiness. Naming that they adhered to the Standards for Reporting Qualitative Research checklist is not sufficient, particularly when asked to provide more evidence.

 Scientifically appropriate would have been a reasoning of why this is not appropriate for this method and how you assured that your interpretation is appropriate and not biased.

 You used  mainly your interpretation of the data for your reasoning. From an academic point of view I would have expected to see a justification in your response based on the method you used or any other scientific reasoning. The provided argument from the authors “overly quote heavy” does not reflect an academic reasoning. Please clarify this to achieve the quality criteria of trustworthiness of the analysis.

 I hope my feedback encourages the authors to improve the paper.

Is the work clearly and accurately presented and does it cite the current literature?

No

If applicable, is the statistical analysis and its interpretation appropriate?

Not applicable

Are all the source data underlying the results available to ensure full reproducibility?

No

Is the study design appropriate and is the work technically sound?

Partly

Are the conclusions drawn adequately supported by the results?

Partly

Are sufficient details of methods and analysis provided to allow replication by others?

No

Reviewer Expertise:

Gerontology, COVID-19 pandemic research, qualitative methodology, risk communication, cross-cultural studies

I confirm that I have read this submission and believe that I have an appropriate level of expertise to confirm that it is of an acceptable scientific standard, however I have significant reservations, as outlined above.

# Reviewer report: Reviewer response for version 2

**DOI:** 10.21956/wellcomeopenres.22906.r71274

**Published:** 2024-02-19

**Authors:** Elfriede Derrer-Merk

**Affiliations:** 1 University of Liverpool, Liverpool, UK

Dear authors, I appreciate your efforts to incorporate the cultural aspects of the African philosophical framework Ubuntu, in the paper. This helps the reader to understand the study much better and strengthens the manuscript.

 As it is challenging to see what the authors changed in a new version without highlighting the changes, I compared the two versions and found that some aspects from the first feedback are missing.

 This is the case in the method section, findings, and discussion. I would stress that the authors are looking at the feedback 1 again and change accordingly.

**Abstract:**

 Please add as suggested the Ubuntu philosophy into the background.

**Method:**

 There is still no clarity for how many participants took part when and how often. The Table you provided as appendix does not give an overview. I suggest to combine the four separate sheets into one table. Please provide one table with all the information.

 I suggested to create one table included into the manuscript which can give an overview. The table in the appendix is not transparent enough. This should include demographics and living situation as you mentioned in the discussion and if you think it helps to add the themes with quotations as suggested in feedback 1.

 If the following source is the evidence of how many participants took part when, then it needs to be clarified in the findings who’s quote is whom and from which interview 1-4 it was taken.

 Underlying data for ‘COVID-19 and older people’s wellbeing in northern KwaZulu-Natal – the importance of relationships’. https://doi.org/10.6084/m9.figshare.19738477 ( Manyaapelo *et al*., 2022a)

 Second, your way of analysis is not clear. You mentioned that you used a framework coding approach and thematic matrix. Please explain in detail what this includes and please provide examples and explain in more detail, as the reader might be interested and would want to learn more about the method you used. The description you provided does not provide transparency of your analytic process. Please see the reference you cited and how they suggest to present the analytical process.

 “A spreadsheet is used to generate a matrix and the data are ‘charted’ into the matrix. Charting involves summarizing the data by category from each transcript. Good charting requires an ability to strike a balance between reducing the data on the one hand and retaining the original meanings and ‘feel’ of the interviewees’ words on the other. The chart should include references to interesting or illustrative quotations.” Gale *et al.* (2013 ^1^). This could be part of the analysis.

 The question why you used different interview points - understood as longitudinal study - is still not answered. This might be challenging as you intended to explore the experience over time, I assume, with a shortfall of 17 participants (how many took part in all 4 interviews). What does this actually mean?

 How did you deal with the short fall and theme saturation? Did you include more participants over time or did you relay on the ones that agreed at the first time?

**The results:**

 The following should go into methods and be more detailed. The provided summary is insufficient and should be more detailed:

 "Eleven men and fifteen women between the ages of 61 and 88 years participated in a total of 87 interviews over a period of four months. It was not possible for all the participants to manage a call every month for four months (which accounts for the shortfall of 17 interviews), but for all those who took part we were able to trace with them their sense of wellbeing across the study months. An anonymized summary of the interviews is available (Manyaapelo *et al*., 2022a)."

 I suggested to revise and to provide examples of your description to clarify and provide evidence for your interpretation. Unfortunately, this is not visible in the 2 ^nd^ version. It is important to make the reader experience the trustworthiness of your analysis. Please make sure you are supporting your arguments with quotations. Please see my feedback in version 1.

 These articles might help:

1. Eldh *et al.* (2020 ^2^).
2. Lingard (2019 ^3^).
3. Sheard (2022 ^4^). 

I would also suggest to strengthen the paper to link the philosophical aspects of Ubuntu with the findings.

**Discussion:**

 The following statement can have a substantial impact on people’s wellbeing and should be transparent in a demographic table and explained why this might impact the participants wellbeing.

 “Most of the participants in this study did not live with their adult children.”

 Overall, the authors did only consider some aspects of the previous feedback related to the manuscript. Please check again and change accordingly.

 The changes the authors made have strengthened the paper but some questions are still open and need to be addressed before the manuscript is ready to be approved.

 I would also suggest to highlight the changes either in the manuscript or comment in detail in the feedback.

 I am looking forward to seeing the suggestion of transparency to gain trustworthiness included into the paper.

Is the work clearly and accurately presented and does it cite the current literature?

No

If applicable, is the statistical analysis and its interpretation appropriate?

Not applicable

Are all the source data underlying the results available to ensure full reproducibility?

No

Is the study design appropriate and is the work technically sound?

Partly

Are the conclusions drawn adequately supported by the results?

Partly

Are sufficient details of methods and analysis provided to allow replication by others?

No

Reviewer Expertise:

Gerontology, COVID-19 pandemic research, qualitative methodology, risk communication, cross-cultural studies

I confirm that I have read this submission and believe that I have an appropriate level of expertise to confirm that it is of an acceptable scientific standard, however I have significant reservations, as outlined above.

## References

[1] : Using the framework method for the analysis of qualitative data in multi-disciplinary health research. *BMC Med Res Methodol* .2013;13: 10.1186/1471-2288-13-117 117 10.1186/1471-2288-13-117 24047204 PMC3848812

[2] : Quotations in Qualitative Studies: Reflections on Constituents, Custom, and Purpose. *International Journal of Qualitative Methods* .2020;19: 10.1177/1609406920969268 10.1177/1609406920969268

[3] : Beyond the default colon: Effective use of quotes in qualitative research. *Perspectives on Medical Education* .2019;8(6) : 10.1007/S40037-019-00550-7 360-364 10.1007/S40037-019-00550-7 PMC690437431758490

[4] : Telling a story or reporting the facts? Interpretation and description in the qualitative analysis of applied health research data: A documentary analysis of peer review reports. *SSM - Qualitative Research in Health* .2022;2: 10.1016/j.ssmqr.2022.100166 10.1016/j.ssmqr.2022.100166

# Response

**Published:** 2024-03-11

**Authors:** Thabang Manyaapelo

**Affiliations:** Africa Health Research Institute, South Africa

Dear authors, I appreciate your efforts to incorporate the cultural aspects of the African philosophical framework Ubuntu, in the paper. This helps the reader to understand the study much better and strengthens the manuscript.

 As it is challenging to see what the authors changed in a new version without highlighting the changes, I compared the two versions and found that some aspects from the first feedback are missing.

 This is the case in the method section, findings, and discussion. I would stress that the authors are looking at the feedback 1 again and change accordingly.

   Dear Elfriede Derrer-Merk, we would like to thank you for taking time to review and for the helpful feedback on our manuscript. As with the first round of feedback, we have made attempts to address each point however where we did/do not agree with your suggestions we responded to that effect. We still maintain that the Ubuntu philosophy is better placed in the Introduction and Discussion since we do not wish for it to steer focus from the wellbeing framework discussed this paper. You seek for clarity on ‘how many participants took part when and how often’ in the methods section of your review and but later you correctly mention that participants took part in 4 interviews’.  As this indicates we had included the information on numbers etc. The detail of our responses are listed below. 

**Abstract:**

 Please add as suggested the Ubuntu philosophy into the background. **Response: **Thank you for the suggestion however we feel that adding anything about the Ubuntu philosophy will steer the reader away from the framework underpinning our analysis of the paper, which is the wellbeing framework. Although the Ubuntu philosophy is key to in helping to explain the context of our participants, we think that it should not be emphasized in the abstract

**Method:**

 There is still no clarity for how many participants took part when and how often. The Table you provided as appendix does not give an overview. I suggest to combine the four separate sheets into one table. Please provide one table with all the information.

 I suggested to create one table included into the manuscript which can give an overview. The table in the appendix is not transparent enough. This should include demographics and living situation as you mentioned in the discussion and if you think it helps to add the themes with quotations as suggested in feedback 1. **Response: **We interviewed a total of 26 people at four time intervals as noted in the Results section. Our target was 104 interviews but we only managed 87 with a shortfall of 17. The quotations and summaries are incomplete deliberately to retain anonymity of the research participants. The informed consent obtained assured participants that we would maintain confidentiality and assured them that we would not use their names or any identifying information.   We presented the data as summaries deliberately to avoid attributing quotations to participants to maintain confidentiality as mentioned above. 

   If the following source is the evidence of how many participants took part when, then it needs to be clarified in the findings who’s quote is whom and from which interview 1-4 it was taken.

 Underlying data for ‘COVID-19 and older people’s wellbeing in northern KwaZulu-Natal – the importance of relationships’. https://doi.org/10.6084/m9.figshare.19738477 ( Manyaapelo *et al*., 2022a)

**Response:**The question of assigning quotations to participants is addressed above. Second, your way of analysis is not clear. You mentioned that you used a framework coding approach and thematic matrix. Please explain in detail what this includes and please provide examples and explain in more detail, as the reader might be interested and would want to learn more about the method you used. The description you provided does not provide transparency of your analytic process. Please see the reference you cited and how they suggest to present the analytical process.

 “A spreadsheet is used to generate a matrix and the data are ‘charted’ into the matrix. Charting involves summarizing the data by category from each transcript. Good charting requires an ability to strike a balance between reducing the data on the one hand and retaining the original meanings and ‘feel’ of the interviewees’ words on the other. The chart should include references to interesting or illustrative quotations.” Gale *et al.* (2013 ^1^ ). This could be part of the analysis. **Response:** We first developed a coding framework which was a combination of the emerging data and topic guide. The next step was developing the analytical memos which were organized along the emerging themes. The thematic matrix is represented by the Excel spreadsheet ( https://doi.org/10.6084/m9.figshare.19738477 ). Wellbeing came out strong from these initial analyses. The seven themes were then further organized by the three dimensions of wellbeing explained by White, 2010. We added the sentence below to the Data analysis section to help explain these steps: Data analysis was a 5-step process, 1) transcription and translation, 2) coding and coding framework, 3) analytical memos, 4) thematic matrix, 5) wellbeing framework orientation. 

 The question why you used different interview points - understood as longitudinal study - is still not answered. This might be challenging as you intended to explore the experience over time, I assume, with a shortfall of 17 participants (how many took part in all 4 interviews). What does this actually mean?

 How did you deal with the short fall and theme saturation? Did you include more participants over time or did you relay on the ones that agreed at the first time?

**Response: **We interviewed a total of 26 people at four time intervals as noted in the Results section. Our target was 104 interviews but we only managed 87 with a shortfall of 17. The shortfall of 17 was not of the participants but of the intended interviews since it was not possible to get hold of every single participants for each of the interview rounds. We did not include more participants over time, we remained with the initial 26 participants. 

**The results:**

 The following should go into methods and be more detailed. The provided summary is insufficient and should be more detailed:

 "Eleven men and fifteen women between the ages of 61 and 88 years participated in a total of 87 interviews over a period of four months. It was not possible for all the participants to manage a call every month for four months (which accounts for the shortfall of 17 interviews), but for all those who took part we were able to trace with them their sense of wellbeing across the study months. An anonymized summary of the interviews is available (Manyaapelo *et al*., 2022a)."

   I suggested to revise and to provide examples of your description to clarify and provide evidence for your interpretation. Unfortunately, this is not visible in the 2 ^nd^ version. It is important to make the reader experience the trustworthiness of your analysis. Please make sure you are supporting your arguments with quotations. Please see my feedback in version 1.

**Response: **The above-mentioned summary cannot be included in the methods section since it summarized the findings.  As stated in the Methods below this is how we arrived at the sample as we presented: The sample was selected randomly by a PIPSA data manager, based on the age criteria and records of access to a mobile telephone. An effort was also made to select people from across the PIPSA area to ensure a spread of people from more and less remote locations. After each of the calls made to the participants, airtime (time paid for a set amount of mobile phone use) would be loaded onto each of the participants mobile phones as a token of appreciation for their time.

 These articles might help:

1. Eldh *et al.* (2020 ^2^ ).
2. Lingard (2019 ^3^ ).
3. Sheard (2022 ^4^ ). 

I would also suggest to strengthen the paper to link the philosophical aspects of Ubuntu with the findings.

**Discussion:**

 The following statement can have a substantial impact on people’s wellbeing and should be transparent in a demographic table and explained why this might impact the participants wellbeing.

 “Most of the participants in this study did not live with their adult children.”

 Overall, the authors did only consider some aspects of the previous feedback related to the manuscript. Please check again and change accordingly. **Response: **Thank you. We incorporated the aspects of the previous feedback that were in line with our intended focus for this manuscript and revised where we could. The editorial process of the journal does not allow for the manuscript to be published in tracked changes therefore we are unable highlight the changes as requested. This why the changes are detailed in the response to the reviewer. Thank you again for the very helpful comments and suggestions in both rounds, these have strengthened this manuscript immensely.    

 The changes the authors made have strengthened the paper but some questions are still open and need to be addressed before the manuscript is ready to be approved.

 I would also suggest to highlight the changes either in the manuscript or comment in detail in the feedback.

 I am looking forward to seeing the suggestion of transparency to gain trustworthiness included into the paper. 

# Reviewer report: Reviewer response for version 1

**DOI:** 10.21956/wellcomeopenres.19754.r65929

**Published:** 2023-09-15

**Authors:** Elfriede Derrer-Merk

**Affiliations:** 1 University of Liverpool, Liverpool, UK

Dear authors, thank you very much for exploring the important aspect of older adult’s well-being related to relationships in KwaZulu-Natal, South Africa. I am convinced that this study is an important contribution not only to the research community but also to the country and people in KwaZulu-Natal itself. The decision to use a qualitative approach adds value to the research, while also enhancing the breadth of coverage for early pandemic-related research.

 However, I have important suggestions for changes to be made before considering the manuscript for indexing.

 The main aspect of the manuscript focuses on intergenerational, diagonal and community relations and the importance of how people are embedded into society but were disrupted to enact their ubuntu values during the pandemic. But what does this mean for the people and researchers?

 Can you make clear what the ubuntu values and ethical framework are and how this is related to wellbeing? As the material, social and individual aspects are drawn from a wellbeing concept.

 I suggest separating these concepts into paragraphs and then finding where they are linked or differ.

 Please provide more detail about the cultural aspects as you mentioned they are crucial to understanding the experiences of older adults in South Africa. What kind of rituals are enacted, and what values are important and could not be lived during the pandemic? You are referring to it throughout the manuscript, but I would suggest explaining it in the introduction, so you can refer to it, rather than giving examples at the discussion.

**Abstract:**

- You mentioned in the title and sequentially in the introduction, results, and discussion the importance of relationships. Please consider this in the abstract.
- I suppose you undertook a longitudinal study, the phrase ‘up to four monthly’ does not clarify how often at which time points you interviewed an x amount of participants. Please clarify this.
- Something is missing ‘and to somewhere they’. Please check.
- You are referring to the wellbeing framework, please mention the Ubuntu framework here.

**Introduction:**

- The following citation is missing in the references - Esther Choo (2020) is not named in the references (Choo, 2020 ^1^).
- I thank the authors for illustrating the lived experiences of the Gogo case.
- The quote includes family living situation, job loss of her son, medical and health conditions, support from grandson, illness and death of family members, funeral challenge, connectivity with church members and missing religious practises.
- As this section provides an example of the broad impact on older adults' lives I would suggest discussing this.
- You are then continuing with aspects of wellbeing and vulnerability related to relationships.
- The aspect of vulnerability of older adults (common stereotype) and the excess death rate needs to be critically explained. You might consider using Baltes and Baltes and/or Carstensen or others.  Please define vulnerability, what are social, psychological etc. vulnerability. Can you explore this with examples, please?
- Why is this sentence relevant? I would suggest deleting it or explaining the importance:
  "The growing literature on the mental health impacts of the pandemic on young people has focused on a different age group, a different generation that are a group at risk (Álvarez-Iglesias *et al*., 2021; Banati *et al*., 2020)."
- What does it mean, what do you want to say here?
  "Yet, when we reflect on the links that exist between people, the focus on specific age groups of people diverts attention away from the relationships between those people, people like Gogo described above, their interdependence, and the ways in which vulnerability and strength may be shared."
- When you refer to the strength of older people, what do you mean, can you explore this and reference it, please?
- I would suggest looking at Antonuccis 2013’s convoy model, how does this differ from the Ubuntu framework? If you are describing the Ubuntu framework at the beginning, this might help you strengthen the argument as to why social connectedness is important for social wellbeing?
- I would suggest having a section where you describe in detail the Ubuntu framework, how it differs from the convoy model and why it is important for the wellbeing of older adults embedded in the Kwazulu-Natal society.

**Ageing and wellbeing:**

- If you want to pursue the concept of wellbeing, please describe the links to relationships and the Ubuntu framework, and why it is important. Please rethink this section.
- If you want to continue with the wellbeing concept then the section ageing and wellbeing needs to include the aspect of purpose in life as this is a very important aspect of wellbeing. This study might help to find the link to the Ubuntu framework: Steger *et al.* (2008 ^2^).   

**Study setting:**

- Please consider the justification of the study. Why have you chosen this area? Please describe the background.
- The study setting provides socio-economic information not to describe socio and culture. Please provide more aspects of the culture, like funerals, celebrating weddings, helping with health care, beliefs and traditions.
- You are talking about it briefly in the discussion, but I would suggest including these examples in the study setting section.
- It could help to put the socio-economic data in a table to make it better readable and then explain the consequences and why you have chosen this area.
- How is this shaped, what does it mean? Can you explain the structures as the reader might like to learn more about them?
  "Although South Africa is a democratic republic, the district is dominated by traditional structures, which inform and shape the local value systems and norms (Beall *et al*., 2005)."

**COVID in South Africa:**

- Please provide more details on what the disaster measures included and how often and long this lasted. This could be demonstrated in a figure with a timeline of disaster measures and data collection.
- You are continuing then with data collection, despite the section being named later. Please re-organise the section.
- I would suggest providing more details of the process of the analyses from codes to categories and themes and how this is related to the Ubuntu wellbeing framework.
- This part should be moved to the method section.

**Method:**

- Please clarify when and how many participants were interviewed and at which point, were they the same?
- What is the second sample, why did you undertake the focus group discussion, and how did this differ from the other sample? Please provide a table with the two different samples and some socio-demographic data like age, gender, living circumstances, distance to the hospital, income (if available) etc. which could give more detail under the specific circumstances. The data you provided are incomplete and do not give an oversight.
- The tables you provided are not clear enough, please combine the four interview times in one table as this makes it easier to read.
- I could not find the focus group socio-demographic table, please add this to the manuscript. I would suggest to integrate the tables into the manuscript.
- I understand you have chosen a longitudinal approach. Please justify why and what your aim was. Please clarify this.

**Data analysis:**

- How was the transcription undertaken, who did it and how was the translation undertaken? Did the person who interviewed participate in the analysis? How did you manage the challenge of keeping the meaning of the original quotes? I would suggest adding a table with the translated quotes you are using in the manuscript.
- The table you provided with some quotes and the summary you provided, is incomplete and the summary does not reflect the associated quote. I would suggest rethinking about it. Your analysis is more likely to be descriptive rather than analytical, please rethink your analysis.
- What analysis method did you use, it seems to be grounded theory, but you mention ‘coded thematically', please provide more details.
- Was your analysis inductive, deductive, or both? (reference it please).
- What is a thematic matrix and can you provide an example? Can you make transparent how you came from codes, and categories to themes, please?
- How did you undertake the analysis from the focus group? Please provide more details.

**Findings:**

- Overall, please provide evidence for your statements. All your arguments need evidence from the participants. Describing the background of your argument helps to understand but does not meet the requirement of evidence-based analysis. I suggest adding more quotes for evidence.
- If you are using grounded theory, I would suggest looking at the meaning, please check Charmaz. If you use another analysis method please draw on this in your analysis. (including referencing it) (this is part of the Method section).
- Were there aspects that did not fit into the framework of wellbeing?
- I could not see the findings from the focus group, are they different or similar, why do they matter?
- I would suggest going back to your findings and thinking carefully if the framework you used fits the data or better what is your data telling you.  
- The following examples might highlight the importance of re-analysing.
- There are inconsistencies in the analysis e.g. the important aspect seems to me not that the participant was sceptical initially, but the official news that people died increased his fear and said he is still very scared. Please refocus on the analysis.
- A 60-year-old man, Sphe, had initially been sceptical about the pandemic in the first few weeks after the news broke. *“I started* *believing it when I started hearing from the radios that people* *are dying and even now my fear is escalating, I am still* *very scared”*.
- What does the quote mean, and what is the participant's experience with it? Was it worth looking for help with the car, whilst the son took his own life?
  "Thabo, was approached by their neighbour to ask if they could use his car to transport their adult child, who was mentally unwell, to the hospital".
- This is a bit confusing, as it is not clear what happened nor why he had the food. Please clarify:
  "This statement suggests that he perceived a state of lacking enough food as his normal situation. The month before we spoke to him, with his family around, they had had enough to eat."
- Did the restrictions allow to visit the church members?
- This sense of community and neighbourliness can also be seen in how some church groups would visit some of their members for prayer and communion. These visitations also served to deliver food parcels to the congregants who could no longer attend church because of the restrictions.
- The importance of relationships during the pandemic is striking, however, have you found that all relationships are equal, or tell your data that the importance is not the number or variety of contact, but how meaningful they were to the participants?

**Discussion:**

- How did your categories link to your findings?
- You are talking in general about social relations like this Social relationships have been found to have a significant impact on health and the manner through which this happens this could go into the introduction.
- I would suggest checking for consistency in your categorisation. You are now talking about the impact of social relations on behavioural, psychosocial and physiological (Umberson & Karas Montez, 2010).
- Did you find any evidence of the cognitive decrease in your study? And why is this relevant to the discussion? It could go into the introduction to highlight the importance of relationships/loneliness and cognitive decline.
- The authors also found that those who were socially isolated and lonely showed increased odds of MCI or dementia compared to those who were socially isolated but not lonely, as well as those who were lonely but not isolated.
- Where are you referring to in this example? Please clarify (name the participant and make sure your discussion is mentioned earlier.
  "This ensures that there is never a parental void in the event a parent, for any number of reasons (away for work, death), is absent. We have noted above the pride and joy an uncle felt in being appreciated by the nephews and nieces, while another man was distraught at not being able to perform his customary role at a relative’s funeral."
- When speaking about the funeral and how people could not enact their traditions, what does this mean for the participants and the society with the ubuntu values? Did people not feel well? What is your data telling you?
- Please name all participants, are the mentioned names origin and did participants agree to be named, or were the names created? (please explain briefly in the method section).
- Please check plain English and the consistency of your citation within the text and in the reference section.
- What is airtime and how much did everyone get? This could go into the method section.
- Overall, the manuscript needs to be restructured, clarified in the methodological approach, and re-analysed. I would like to encourage the authors to provide transparency in the whole research process.
- You could use the CASP tool to check the transparency of your revised manuscript (Long *et al.*, 2020 ^3^).

I am looking forward seeing the suggested changes included in the manuscript.

Is the work clearly and accurately presented and does it cite the current literature?

No

If applicable, is the statistical analysis and its interpretation appropriate?

Not applicable

Are all the source data underlying the results available to ensure full reproducibility?

No

Is the study design appropriate and is the work technically sound?

Partly

Are the conclusions drawn adequately supported by the results?

Partly

Are sufficient details of methods and analysis provided to allow replication by others?

No

Reviewer Expertise:

Gerontology, COVID-19 pandemic research, qualitative methodology, risk communication, cross-cultural studies

I confirm that I have read this submission and believe that I have an appropriate level of expertise to state that I do not consider it to be of an acceptable scientific standard, for reasons outlined above.

## References

[1] : COVID-19 fault lines. *Lancet* .2020;395(10233) : 10.1016/S0140-6736(20)30812-6 1333 10.1016/S0140-6736(20)30812-6 32334693 PMC7180038

[2] : Being good by doing good: Daily eudaimonic activity and well-being. *Journal of Research in Personality* .2008;42(1) : 10.1016/j.jrp.2007.03.004 22-42 10.1016/j.jrp.2007.03.004

[3] : Optimising the value of the critical appraisal skills programme (CASP) tool for quality appraisal in qualitative evidence synthesis. *Research Methods in Medicine & Health Sciences* .2020;1(1) : 10.1177/2632084320947559 31-42 10.1177/2632084320947559

# Response

**Published:** 2023-12-05

**Authors:** Thabang Manyaapelo

**Affiliations:** Africa Health Research Institute, South Africa

Dear Elfriede Derrer-Merk, Thank you for your comments.  We have revised our paper accordingly and respond to many of the comment below.  We have assigned a lose numbering system to your comments to make this response easier to follow.  Where we refer to page numbers, we are referring to the track changed version of the paper.  

1. Dear authors, thank you very much for exploring the important aspect of older adult’s well-being related to relationships in KwaZulu-Natal, South Africa. I am convinced that this study is an important contribution not only to the research community but also to the country and people in KwaZulu-Natal itself. The decision to use a qualitative approach adds value to the research, while also enhancing the breadth of coverage for early pandemic-related research.

  However, I have important suggestions for changes to be made before considering the manuscript for indexing.   The main aspect of the manuscript focuses on intergenerational, diagonal and community relations and the importance of how people are embedded into society but were disrupted to enact their ubuntu values during the pandemic. But what does this mean for the people and researchers? Can you make clear what the ubuntu values and ethical framework are and how this is related to wellbeing? As the material, social and individual aspects are drawn from a wellbeing concept. I suggest separating these concepts into paragraphs and then finding where they are linked or differ.   Please provide more detail about the cultural aspects as you mentioned they are crucial to understanding the experiences of older adults in South Africa. What kind of rituals are enacted, and what values are important and could not be lived during the pandemic? You are referring to it throughout the manuscript, but I would suggest explaining it in the introduction, so you can refer to it, rather than giving examples at the discussion.  

Response:  Thank you – your comments made us realise that we had had not explained Ubuntu properly for readers unfamiliar with African philosophy.  We provide detail on our response on this below.    

**2. Abstract:** You mentioned in the title and sequentially in the introduction, results, and discussion the importance of relationships. Please consider this in the abstract.

Response: The abstract was revised to reflect this suggestion. Please see page 1.

 ○ 3. I suppose you undertook a longitudinal study, the phrase ‘up to four monthly’ does not clarify how often at which time points you interviewed an x amount of participants. Please clarify this.

Response:  We have rewritten this.  

 4. Something is missing ‘and to somewhere they’. Please check.

Response: This has been corrected, thank you.  

 5. You are referring to the wellbeing framework, please mention the Ubuntu framework here. Response: The study uses the wellbeing framework to guide the analysis of the findings. The concept of Ubuntu was introduced to put the findings into an African context and to explain the importance of interconnectedness.  It was not intended as a framework  for the analysis therefore we have not included it in the abstract.   As we note above, your comments made it clear to us that the concept of Ubuntu may not be familiar to people from outside sub-Saharan Africa, so we have explained the concept in more detail in the paper. Please see page 6.  

**6. Introduction:** ○ The following citation is missing in the references - Esther Choo (2020) is not named in the references (Choo, 2020).

Response: This was already included in the references.

   7.  I thank the authors for illustrating the lived experiences of the Gogo case. ○ The quote includes family living situation, job loss of her son, medical and health conditions, support from grandson, illness and death of family members, funeral challenge, connectivity with church members and missing religious practices. ○ As this section provides an example of the broad impact on older adults' lives I would suggest discussing this. ○ You are then continuing with aspects of wellbeing and vulnerability related to relationships. ○ The aspect of vulnerability of older adults (common stereotype) and the excess death rate needs to be critically explained. You might consider using Baltes and Baltes and/or Carstensen or others.  Please define vulnerability, what are social, psychological etc. vulnerability. Can you explore this with examples, please?

Response:  We have provided a definition of vulnerability – focusing on the concept of `situations of vulnerability’ from Levasseur et al. (2022) which we find helpful when thinking about the combination of factors which contributed to vulnerability during the pandemic. This takes the emphasis away from an individual to look at the combination of circumstances which may increase the risk of harm for a person or groups of people.  During the pandemic older people were in a situation of vulnerability not only because they were more susceptible to severe disease than younger people, but also because of the isolation caused by needing to stay at home and being preventing from meeting friends and attending church and other places they were used to meeting other people.    

 8. Why is this sentence relevant? I would suggest deleting it or explaining the importance: "The growing literature on the mental health impacts of the pandemic on young people has focused on a different age group, a different generation that are a group at risk (ÁlvarezIglesias *et al*., 2021; Banati *et al*., 2020)."

Response:  We have deleted that sentence.  

 9. What does it mean, what do you want to say here? "Yet, when we reflect on the links that exist between people, the focus on specific age groups of people diverts attention away from the relationships between those people, people like Gogo described above, their interdependence, and the ways in which vulnerability and strength may be shared." Response:  We have rewritten this sentence.  This sentence connects to the information on linked lives above, but also leads into the information on Ubuntu which follows.  

 10. When you refer to the strength of older people, what do you mean, can you explore this and reference it, please?

Response: We have now included a definition for strength on page 4.  

 11. I would suggest looking at Antonuccis 2013’s convoy model, how does this differ from the Ubuntu framework? If you are describing the Ubuntu framework at the beginning, this might help you strengthen the argument as to why social connectedness is important for social wellbeing?

Response: Thank you.  While the Convoy model is a very useful model that seeks to integrate research conducted on social relations, and has been used to understand the predictors and consequences of social relations across the life course, it is not a substitute for our explanation of Ubuntuism. As we explain above the concept of Ubuntu is taken from African philosophy. Ubuntu is therefore the manner in which peoples can be seen to interact with the world, we do not use it as a theoretical framework in this paper.  

 12. I would suggest having a section where you describe in detail the Ubuntu framework, how it differs from the convoy model and why it is important for the wellbeing of older adults embedded in the Kwazulu-Natal society.

Response: We have not used the Convoy model, as explained above it is not a substitute for Ubuntu. The concept of Ubuntu is now explained in more detail.  

**13. Ageing and wellbeing:** If you want to pursue the concept of wellbeing, please describe the links to relationships and the Ubuntu framework, and why it is important. Please rethink this section.  ○ If you want to continue with the wellbeing concept then the section ageing and wellbeing needs to include the aspect of purpose in life as this is a very important aspect of wellbeing. This study might help to find the link to the Ubuntu framework: Steger *et al.* (2008 ^2^).  

Response: We agree that purpose of life is an important aspect to consider when investigating wellbeing. We have however not used it for this study since our objective was to investigate wellbeing through the lens of the wellbeing framework explained on page 7.  

**14. Study setting:** Please consider the justification of the study. Why have you chosen this area? Please describe the background.

Response: We conducted this study to document the experiences of older people during the COVID-19 pandemic lockdowns and the impact these mandatory restrictions had on their wellbeing. This area was purposely chosen because the Africa Health Research Institute has been working with this community for over 20 years. The study was intended to explore possible avenues that could lead to intervention development aimed at improving living conditions for older people in this community. We have clarified this is on page 11.  

 15. The study setting provides socio-economic information not to describe socio and culture. Please provide more aspects of the culture, like funerals, celebrating weddings, helping with health care, beliefs and traditions.

Response:  We had included some additional information on this on page 9.  

 16. You are talking about it briefly in the discussion, but I would suggest including these examples in the study setting section. It could help to put the socio-economic data in a table to make it better readable and then explain the consequences and why you have chosen this area.  How is this shaped, what does it mean? Can you explain the structures as the reader might like to learn more about them? "Although South Africa is a democratic republic, the district is dominated by traditional structures, which inform and shape the local value systems and norms (Beall *et al*., 2005)."

Response: We have added the text to explain traditional structures on page 9.    

**17. COVID in South Africa:** Please provide more details on what the disaster measures included and how often and long this lasted. This could be demonstrated in a figure with a timeline of disaster measures and data collection.

Response: Thank you for the suggestion. We have provided additional information on the response to COVID-19 on page 10.     

 18. You are continuing then with data collection, despite the section being named later. Please re-organise the section.

Response:  This is included here because it is situating the timing of our data collection relative to the lockdown restriction measures.  

 19 I would suggest providing more details of the process of the analyses from codes to categories and themes and how this is related to the Ubuntu wellbeing framework. This part should be moved to the method section.

Response: The process of the analysis is described on page 14 and 15 in the Methods section.  As we have already explained we have not used Ubuntu as a framework.  

**20. Method:** Please clarify when and how many participants were interviewed and at which point, were they the same?

Response:  This information had been included at the beginning of the findings on the previous version – please see page 15.  We have augmented this information to make it clearer.  

 21. What is the second sample, why did you undertake the focus group discussion, and how did this differ from the other sample? Please provide a table with the two different samples and some socio-demographic data like age, gender, living circumstances, distance to the hospital, income (if available) etc. which could give more detail under the specific circumstances. The data you provided are incomplete and do not give an oversight. Response:  We realise mention of the focus group is confusing, so we have removed that from this paper, since those data are not used here.  

 22. The tables you provided are not clear enough, please combine the four interview times in one table as this makes it easier to read.

Response:  We assume this is in reference to the supplementary file. We find this approach allows the user to see the examples more easily than have one very large file.     

 23. I understand you have chosen a longitudinal approach. Please justify why and what your aim was. Please clarify this.

Response: The longitudinal approach was used to see how the lockdown restrictions impacted on the wellbeing of older people over the duration of mandatory restrictions since normal services had been interrupted.  

**24. Data analysis:** How was the transcription undertaken, who did it and how was the translation undertaken? Did the person who interviewed participate in the analysis? How did you manage the challenge of keeping the meaning of the original quotes? I would suggest adding a table with the translated quotes you are using in the manuscript.

Response: The translations were conducted by the same people who conducted the interviews. This has been revised on page 14.  

 25. The table you provided with some quotes and the summary you provided, is incomplete and the summary does not reflect the associated quote. I would suggest rethinking about it. Your analysis is more likely to be descriptive rather than analytical, please rethink your analysis.

Response: As we say above, the quotations and summaries are incomplete deliberately to retain anonymity of the research participants. The informed consent obtained assured participants that we would maintain confidentiality and assured them that we would not use their names or any identifying information.   

 26. What analysis method did you use, it seems to be grounded theory, but you mention ‘coded thematically', please provide more details. ○ Was your analysis inductive, deductive, or both? (reference it please). ○ What is a thematic matrix and can you provide an example? Can you make transparent how you came from codes, and categories to themes, please? Response: The thematic analysis is explained in page 14 and 15 and we have expanded our explanation on the approach to our analysis in that section.  

 27.  How did you undertake the analysis from the focus group? Please provide more details. Response:  As noted above, this has been deleted.  

**28. Findings:** Overall, please provide evidence for your statements. All your arguments need evidence from the participants. Describing the background of your argument helps to understand but does not meet the requirement of evidence-based analysis. I suggest adding more quotes for evidence.

Response: We respectfully disagree – and generally in much of what we write prefer a combination of paraphrasing to bring out the points in what people say with quotations to illustrate where helpful.  This provides evidence of the points we make without making a paper overly quote heavy.  

 29. If you are using grounded theory, I would suggest looking at the meaning, please check Charmaz. If you use another analysis method please draw on this in your analysis. (including referencing it) (this is part of the Methods)

Response: We did not use grounded theory – we had a predefined research question, and the topic guides were framed before data collection to explore that question.  This shaped the data collection.  We are very familiar with the work of Charmaz, Glazer and Strauss – we did not, in Kathy Charmaz’s words, `construct theories from the data themselves…’ we went into the data collection with a specific focus to find out about the impact of the COVID-19 restrictions on older people’s lives.  Yes, we gained additional insights into the topic from the data as in recognising `wellbeing’ as an overarching theme, through inductive analysis, but this is very different from a classic grounded theory approach because it augmented the deductive approach.  

 30. Were there aspects that did not fit into the framework of wellbeing?

Response. No we did not have any aspects that did not fit into the framework, all the emerging themes were categorized under the three dimensions of the framework for wellbeing.  

 31. I would suggest going back to your findings and thinking carefully if the framework you used fits the data or better what is your data telling you.   ○ The following examples might highlight the importance of re-analysing.  ○ There are inconsistencies in the analysis e.g. the important aspect seems to me not that the participant was sceptical initially, but the official news that people died increased his fear and said he is still very scared. Please refocus on the analysis.  ○ A 60-year-old man, Sphe, had initially been sceptical about the pandemic in the first few weeks after the news broke. *“I started believing it when I started hearing from the radios that people are dying and even now my fear is escalating, I am still very scared”*.  ○ What does the quote mean, and what is the participant's experience with it? Was it worth looking for help with the car, whilst the son took his own life? "Thabo, was approached by their neighbour to ask if they could use his car to transport their adult child, who was mentally unwell, to the hospital". ○ This is a bit confusing, as it is not clear what happened nor why he had the food. Please clarify: "This statement suggests that he perceived a state of lacking enough food as his normal situation. The month before we spoke to him, with his family around, they had had enough to eat." ○ Did the restrictions allow to visit the church members? ○ This sense of community and neighbourliness can also be seen in how some church groups would visit some of their members for prayer and communion. These visitations also served to deliver food parcels to the congregants who could no longer attend church because of the restrictions.  The importance of relationships during the pandemic is striking, however, have you found that all relationships are equal, or tell your data that the importance is not the number or variety of contact, but how meaningful they were to the participants?

Response:  Thank you for all your suggestions – we have provided some additional information to clarify the text on the points you raise.  We are not going to reanalyse our data.

**32. Discussion:** How did your categories link to your findings? ○ You are talking in general about social relations like this Social relationships have been found to have a significant impact on health and the manner through which this happens this could go into the introduction. ○ I would suggest checking for consistency in your categorisation. You are now talking about the impact of social relations on behavioural, psychosocial and physiological (Umberson & Karas Montez, 2010).

Response: We have provided some additional details in the discussion.  The categorizations explain the three domains of material, relational and subjective which wellbeing is defined under. The behavioural, psychological and physiological aspects are not separate from this definition. Our data are organized to reflect these three domains and also show how relationships cut across there three domains.  

 33. Did you find any evidence of the cognitive decrease in your study? And why is this relevant to the discussion? It could go into the introduction to highlight the importance of relationships/loneliness and cognitive decline. The authors also found that those who were socially isolated and lonely showed increased odds of MCI or dementia compared to those who were socially isolated but not lonely, as well as those who were lonely but not isolated. Response: This study did not measure any cognitive impairment. We are making the argument that social isolation can have an impact on measurable cognitive attributes as demonstrated by the article we cited. This then links back to the definition of wellbeing that includes material, relational and subjective components. We further argue that relationships cut across all three components of this definition. Our findings show that the relationships people had an impact on their state of wellbeing.  

 34. Where are you referring to in this example? Please clarify (name the participant and make sure your discussion is mentioned earlier. "This ensures that there is never a parental void in the event a parent, for any number of reasons (away for work, death), is absent. We have noted above the pride and joy an uncle felt in being appreciated by the nephews and nieces, while another man was distraught at not being able to perform his customary role at a relative’s funeral."

Response: We have added some additional information to the text to clarify.  

 35. When speaking about the funeral and how people could not enact their traditions, what does this mean for the participants and the society with the ubuntu values? Did people not feel well? What is your data telling you?

Response: We hope that the additional information we have provided on Ubuntu makes this clearer.  Given the sense of being a part of a much larger whole – connected to both the living and the dead.  We found that some of our participants were distressed by the inability to perform some of their cultural practices through which they could show respect to those who have died.   

 36. Please name all participants, are the mentioned names origin and did participants agree to be named, or were the names created? (please explain briefly in the method section). Response: Where names are used in this article they are pseudonyms.  This is stated in the Methods > Ethical Approval section.  

 37. Please check plain English and the consistency of your citation within the text and in the reference section.

Response: The citations have been checked as suggested, thank you.  

 38. What is airtime and how much did everyone get? This could go into the method section. Response:  Airtime is the mobile phone voice calling minutes which people access by paying for vouchers to load. We realise many people in the Global North may have mobile phone contracts so not worry about loading airtime – hence this is a term which is not in common usage.  We have explained the term.  

 39. You could use the CASP tool to check the transparency of your revised manuscript (Long *et al.*, 2020 ^3^).

Response: We used the Standards for Reporting Qualitative Research checklist tool, please see in Methods > Data Analysis
